# Evaluating the Nutritional Content of Children’s Breakfast Cereals in Australia

**DOI:** 10.3390/children5070084

**Published:** 2018-06-21

**Authors:** Terence Tong, Anna Rangan, Luke Gemming

**Affiliations:** Nutrition and Dietetics Group, School of Life and Environmental Sciences, Charles Perkins Centre, The University of Sydney, Sydney NSW 2006, Australia; hton1312@uni.sydney.edu.au (T.T.); Anna.Rangan@sydney.edu.au (A.R.)

**Keywords:** breakfast cereals, children, marketing, nutritional content

## Abstract

Breakfast is an important contributor to the daily dietary intake of children. This study investigated the nutritional composition of ready to eat (RTE) children’s breakfast cereals, which display fictional cartoon characters and themes, compared to other cereals available in Australia. Nutrient content claims on packaging were also examined. Data were collected from RTE breakfast cereal packages (*N* = 347) from four major supermarkets in Sydney. Cereals were classified based on product type and promotional information displayed. Overall, 46% of children’s cereals were classified as “less healthy” as per nutrient profiling score criteria. Children’s cereals had a similar energy and sodium content per 100 g compared to other cereals but contained significantly higher levels of total sugar and lower levels of protein and dietary fibre compared to other varieties. Children’s cereals with nutrient content claims had improved (lower) nutrient profiling scores than those that did not (2 vs. 13, *p* = 0.021), but total sugar per 100 g was similar: 25 g (interquartile range (IQR) 14 g) vs. 32 g (IQR 19 g). In conclusion, RTE children’s breakfast cereals were found to be less healthy compared to other cereals on the market and the use of nutrient content claims on children’s cereals may mislead consumers regarding their overall nutrient profile.

## 1. Introduction

Eating a healthy breakfast improves nutritional intakes and has been associated with long term health benefits such as lower risk of overweight and obesity, and reduced risk factors for cardiovascular disease compared with skipping breakfast [1,2]. The most recent national nutrition survey in Australia indicated that 47% of children aged 2–18 years old reported eating breakfast cereals, and one in six children ate “ready to eat” (RTE) pre-sweetened cereals containing more than 15 g sugar per 100 g [3]. Pre-sweetened cereals contributed significantly higher total and free sugars to the daily nutrient intakes but less protein and dietary fibre compared with minimally sweetened cereals [3]. 

Analysis of the survey undertaken by the Australian Bureau of Statistics [4] also revealed that children were not meeting the dietary guideline recommendations to ensure adequate intake of food groups and key nutrients required for optimal health and growth [5]. For example, 67% of children did not meet the minimum serves of grains and cereals and approximately 75% of children consumed in excess of the World Health Organization (WHO) recommendation for free sugar intake [4,6,7,8]. Increasing the consumption of healthy cereal-based breakfasts among children could improve nutritional intake and help meet the dietary guidelines. Daily breakfast consumption has been shown to improve daily nutritional profile, lower the risk of being overweight or obese, and improve cognitive functioning in children compared to non-consumption of breakfast [9,10,11,12].

However, high-sugar breakfast cereals are one of the most promoted products in the world [13], with 54% of products carrying some type of nutrition function or health related claim in Australia [14]. In Australia, products can display front of pack nutrient content claims, general health or high-level health claims if they meet the nutrient profiling score (NPS) and other nutrient specific criteria proposed by Food Standards Australia New Zealand (FSANZ) [15]. General health claims refer to a substance in a food and its effect on health, whereas high-level health claims refer to the food and its relationship with a serious disease or biomarker of a serious disease. There has been an increased effort by the food industry to promote breakfast foods towards children, especially high-sugar RTE breakfast cereals [16,17]. Examples of promoting cereal consumption include television advertisements during children’s programs [18,19], brand mascots (e.g., Kellogg’s Tony the Tiger), and cartoon media to influence children’s choices [20], as well as displaying nutrient content and health claims on breakfast cereal packaging.

This current analysis aimed to compare the nutritional composition of RTE breakfast cereals marketed towards children, with those that are not, as well as assess the association between the presence of nutritional claims and nutritional content.

## 2. Materials and Methods

Nutritional composition data were collected between March and April of 2017 for all breakfast cereals available from the four major supermarkets across Sydney (Coles, Woolworths, Aldi, and IGA) which represent 91% of the market share in Australia [21]. Images were systematically captured of the front, back, nutrition information panel (NIP), ingredients list, and barcodes found on the food packaging with Lenovo Moto G4 smart phones (Lenovo, Quarry Bay, Hong Kong). Images were backed up to a secure network storage at The University of Sydney. Brand name, product title, packaging size, recommended serving size, health star rating (HSR), and nutrients listed on the NIP (energy, protein, total fat, saturated fat, carbohydrate, total sugar, dietary fibre, and sodium), and the presence of nutrient content, or health claims, or both, were recorded on a spreadsheet. Data cleaning was carried out, which included screening and removal of duplicate products. For products which contained different packaging sizes (290 g vs. 805 g cereal boxes), only the product that exhibited the packaging weight closest to the median of that cereal category was included.

Breakfast cereals were classified as RTE if they were able to be consumed without using any cooking techniques, such as boiling and microwaving. In order to distinguish between RTE breakfast cereals that were marketed to children from those that were not, the following inclusion criteria were established: (1) the presence of cartoon or fantasy characters or themes, brand mascots, or celebrities; (2) childhood themes such as sport characters, shapes, and colours; (3) printed statements on the packaging such as “for children” or ”for little kids”; or (4) marketed to a specific age limit or range.

Nutrient profiling scores for all cereals were obtained through an online calculator using the formula provided by FSANZ (Standard 1.2.7) [22,23]. Nutrient profiling score = baseline points − modifying points, where Baseline points included energy (kJ) + saturated fats (g) + sugars (g) + sodium (mg) per 100 g; and modifying points included the percentage of vegetable and fruit content (V points) + protein (P points) + dietary fibre (F points).

A product was deemed to meet the nutrient profiling criterion if the total score was less than four and hence eligible to carry health claims. For the purpose of this study, eligible products (<4) were classified as “healthy”, whilst non-eligible products (≥4) were deemed “less healthy”.

For comparison, RTE breakfast cereals were split into pre-defined categories [24]: “flakes, bubbles, and puffs” (e.g., Cornflakes), “biscuits and bites” (e.g., Weetbix), “brans” (e.g., All-Bran), “muesli” (e.g., Natural Muesli), “clusters and granola” (e.g., Crispy Oat Clusters) and “children’s cereals” (e.g., Milo). Children’s cereals were further grouped according to the presence of nutrient and health claims—“High dietary fibre claim”, “Wholegrains claim”, and “B vitamins claim” (contained any combination of B vitamins including thiamin, riboflavin, niacin, vitamin B6, or folic acid), or “Any nutrient or health claim” (contained any type of nutrient content or health claim such as low glycaemic index, low fat, low sugar, high protein, high dietary fibre, wholegrain, or the presence of any vitamins and minerals).

Data were analysed using SPSS Version 24.0 for Windows (IBM Corporation, New York, NY, USA) using non-parametric statistics due to the results of normality testing (Shapiro–Wilk test) and overall small sample sizes. Data were presented as median and interquartile range (IQR) and the Mann–Whitney *U* test was used to compare the nutrient content, HSR, and NPS between children’s cereals and other cereal categories. A *p*-value < 0.05 was considered significant. Cereals with missing nutrient values (e.g., dietary fibre) or HSRs were excluded from the comparison of that specific variable. Furthermore, cereals that had missing nutrient values required to calculate the profiling score (energy, saturated fat, sugars, protein, sodium, or dietary fibre) were removed from the analysis. Similarly, for children’s cereals with and without nutrient or health claims, the Mann–Whitney *U* tests were undertaken to compare nutrient composition as well as the NPS and HSR.

## 3. Results

Out of 504 breakfast cereal products collected, from which 391 were classified as RTE cereals, 33 were marketed to children. Within this group, 17 had some form of cartoon or fantasy character on the packaging, 14 exhibited childhood themes, and two specifically stated the product was for children. For the cereals marketed to children, the majority contained some type of nutrient content claim (*n* = 25/33) but no claims related to general health were found. A nutritional comparison of these breakfast cereals including NPS and HSR are shown in Table 1. Compared to all other RTE breakfast cereal categories, “children’s cereals” were significantly lower in protein and dietary fibre and significantly higher in total sugar (excluding brans) but there was no consistent trend for energy and sodium. Fat and saturated fat content were generally low in all breakfast cereals except for “muesli” and “clusters and granola”. The combined nutrient differences in “children’s cereals” were reflected in a significantly higher median NPS (indicating less healthy) and significantly lower median HSR compared with all other cereal categories. A comparison to products with missing fibre values did not show any clear differences in nutrient profile among the categories. 

**Figure 1:**
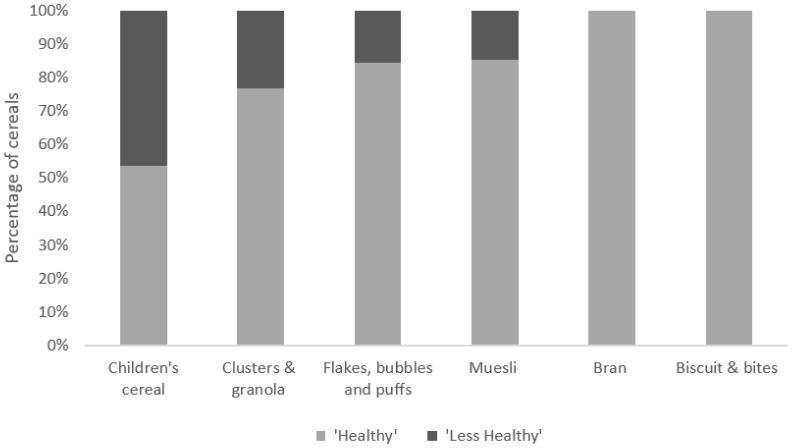
Percentage of Australian breakfast cereals classified as “healthy” and “less healthy”. Nutrient profiling scores were calculated using the formula provided by Food Standards Australia New Zealand (Standard 1.2.7) for “children’s cereals” (*n* = 28), “clusters and granola” (*n* = 56), “flakes, bubbles, and puffs” (*n* = 103), “muesli” (*n* = 109), “bran” (*n* = 7) and “biscuits and bites” (*n* = 18). Products were classified as “healthy” if they received a NPS less than four, whilst products with a NPS score greater or equal to four were deemed “less healthy”.

Figure 1 compares the profiling scores between “children’s cereals” and other categories in more detail. The distribution between “healthy” (NPS < 4) and “less healthy” (NPS ≥ 4) cereals varied across the different cereal types. “Brans” and “biscuits and bites” were the only categories with 100% of products classified as “healthy”. “Children’s cereals” had the lowest proportion (54%) of products classified as healthy compared to all other categories. 

Table 2 details the differences within the “children’s cereals” category between those that contained nutrient content claims versus those that did not. Overall, “children’s cereals” with “any claim” had a significantly lower NPS and higher HSR, mostly as a result of higher dietary fibre and lower sodium. However, 35% of the “children’s cereals” with “any claim” were classified as less healthy. Children’s cereals with wholegrain claims and high dietary fibre claims had significantly higher dietary fibre content and lower NPS. For cereals with B vitamins claims, there were no significant differences for most nutrients (excluding total fat and saturated fat) and no differences in the NPS and HSR. 

## 4. Discussion

This analysis of over 500 currently available RTE breakfast cereals found that cereals marketed to children had inferior nutritional profiles compared to products not marketed to children. Half of children’s cereals did not meet the “healthy” criteria when a nutrient profile score was applied, with children’s cereals containing significantly higher levels of sugar but lower levels of dietary fibre and protein. The presence of a nutrient content claim on children’s cereals was associated with an improved nutrient profiling score, although total sugar per 100 g did not differ.

The sugar content of children’s breakfast cereals remains a major concern. Compared to all other RTE breakfast cereal categories, children’s cereals were significantly higher in sugar and alarmingly, a substantial proportion of the sugar was in the form of added sugars, evident from the ingredients lists with sugar frequently listed as the second or third ingredient. The 2011–2012 Australian Health Survey classified breakfast cereals as discretionary foods if they contained more than 30 g of sugar per 100 g [4]. Applying that criteria to this study, almost one third of children’s cereals would be classified as discretionary with many others close to the cut-point. Some might argue that consuming breakfast cereals high in sugar may increase children’s breakfast consumption and is better than skipping breakfast all together. However, research has shown that children will eat and enjoy low-sugar breakfast cereals when offered, and in more appropriate portion sizes and with more fruit compared with eating high-sugar cereals [25]. In contrast, the levels of protein and dietary fibre were significantly lower in children’s cereals which, if increased, could assist with satiety and reduce overconsumption of high-sugar RTE breakfast cereals [25]. The lower protein and dietary fibre content may be attributed to different cereal grains and lower amounts of nuts and seeds in children’s cereals [26,27].

Findings of the present study are consistent with previous research investigating children’s cereals [28,29,30,31]. Devi et al. analysed the nutritional differences between children’s and other cereals in New Zealand and found that there was a greater percentage of “less healthy” options for cereals marketed towards children [28]. Similarly, a study in the United States found that child-targeted cereals were less nutritious than adult-targeted varieties and had more added sugar sources listed in the first five ingredients [29].

Three quarters of all children’s cereals in our study displayed a nutrient content claim. Not surprisingly, the majority of the nutrient content claims related to the increased levels of specific beneficial nutrients (e.g., dietary fibre), and led to better NPS and HSR, with the exception of cereals carrying B vitamins claims. However, in most cases, the energy and sugar content were not significantly different between the groups. A recent study showed similar results, where the nutritional composition of breakfast cereals with and without claims were reasonably similar with the exception of certain nutrients, such as sodium content [32]. Hence, the presence of nutrient content claims could be misleading to consumers, and especially parents, giving the perception that these foods are healthy when they may not be [33]. Considering that approximately half (46%) of the children’s cereals were classified as “less healthy” in the present study, and 35% of these cereals contained some form of nutrient content claim, the concept of restricting nutrient content claims to products classified as “less healthy” according to the NPS [22] (similar to health claims) should be considered.

The heavy marketing of these products by the food industry undermines the national dietary guidelines [5]. In addition to the various nutrient content claims which are used to persuade consumers to purchase products, cartoon characters and themes are regularly used. Of the 33 varieties of children’s cereals, the majority of them were promoted through the use of cartoon characters and themes (*n* = 31), which have been shown to significantly increase consumption levels in children [34]. Research has shown that children as young as two years of age possess the ability to identify characters and brands on packaging, hence influencing their food selections [35,36]. Television advertising of children’s cereals occurs during peak children viewing sessions such as Saturday morning cartoons [37]. Significantly more promotional characters are shown during food advertisements in children’s peak viewing sessions compared to non-peak times in order to influence children’s food choices [38]. Not surprisingly, increased exposure to such advertisements is positively correlated to their consumption [39]. Hence, more restriction and regulation of these marketing techniques should be implemented for “less healthy” children’s cereals, such as TV advertising, in order to limit their exposure and consumption [39,40].

The strengths of the study included the collection of a large sample of RTE breakfast cereal products, with audited supermarkets representing 92% of the market share. All comparisons were analysed based on nutrients per 100 g, as declared serving sizes on the packages are not standardised and may not represent the amount that consumers eat. Several limitations must be acknowledged. The analysis only takes into account the nutrients available from the RTE cereals on their own, whereas in reality they are consumed with different types of foods, beverages, and condiments such as milk, yoghurt, sugar, honey, fruit, and fruit juice. Furthermore, nutrient information was obtained directly from the NIP of food packages, not from laboratory analysis, thus accuracy was dependent on the manufacturers’ reported data. Although some sample sizes were small, statistical power was sufficient to detect meaningful statistical differences for the main analyses.

## 5. Conclusions

In conclusion, this study highlights important differences in the nutritional quality of RTE breakfast children’s cereals. Overall, children’s cereals were less nutritious, with significantly less protein and dietary fibre content and significantly higher sugar content, compared to other categories. The difference is concerning considering the promotion and advertising of these children’s cereal products. Efforts should be made by food manufacturers to improve the nutrition profile of children’s cereals.

## Figures and Tables

**Table 1:** Comparison of nutrient content between children’s cereals and other ready to eat (RTE) breakfast cereal.

	Children’s Cereals	Flakes, Bubbles and Puffs		Muesli		Clusters and Granola		Brans		Biscuits and Bites	
	*n* = 33	*n* = 114		*n* = 116		*n* = 59		*n* = 7		*n* = 18	
Nutrient/100 g	Median (IQR)	Median (IQR)	*p*-Value	Median (IQR)	*p*-Value	Median (IQR)	*p*-Value	Median (IQR)	*p*-Value	Median (IQR)	*p*-Value
Energy (kJ)	1590 (65)	1570 (783)	0.36	1655 (278)	0.007	1770 (140)	<0.001	1370 (110)	<0.001	1490.0 (113)	0.01
Protein (g)	7.2 (2.5)	8.4 (3.9)	0.001	10.7 (2.6)	<0.001	10.6 (2.7)	<0.001	12.7 (3.2)	<0.001	10.6 (2.8)	<0.001
Fat (g)	1.4 (1.5)	2.6 (3.2)	<0.001	10.4 (8.7)	<0.001	13.4 (5.9)	<0.001	3.9 (2.6)	0.002	1.7 (2.8)	0.048
Saturated fat (g)	0.4 (4.9)	0.5 (0.7)	0.31	2.2 (1.8)	<0.001	2.4 (2.2)	<0.001	0.9 (0.6)	0.095	0.4 (1.5)	0.2
Carbohydrate (g)	80.0 (12.5)	71.5 (14.4)	<0.001	57.5 (7.8)	<0.001	61.0 (7.7)	<0.001	46.8 (20.7)	<0.001	67.0 (3.9)	<0.001
Sugars (g)	26.9 (15.8)	17.0 (14.3)	<0.001	16.3 (7.7)	<0.001	18.6 (3.3)	<0.001	17.7 (10.3)	0.17	5.7 (8.5)	<0.001
Sodium (mg)	254 (245)	250 (237)	0.98	17 (39)	<0.001	60 (123)	<0.001	360 (118)	0.22	270 (100)	0.66
Dietary fibre (g)	5.6 (6.6) ^a^	8.0 (6.5) ^a^	0.004	8.8 (2.9) ^a^	<0.001	7.8 (1.7) ^a^	0.017	29.5 (18.1)	<0.001	10.5 (2.4)	<0.001
Nutrient profiling score	3.0 (13.0) ^b^	1.0 (3.0) ^b^	0.005	0.0 (4.0) ^b^	0.001	1.0 (3.0) ^b^	0.82	0.0 (2.0)	0.199	−3.0 (4.0)	<0.001
Health star rating	3.5 (2.0) ^c^	4.0 (0.5) ^c^	<0.001	4.25 (0.5) ^c^	<0.001	4.0 (0.0) ^c^	0.001	5.0 (0.5)	<0.001	4.5 (1.0)	<0.001

IQR, interquartile range. ^a^ Dietary fibre information was unavailable for 5 children’s cereals, 12 flakes, bubbles and puffs, 7 mueslis, 3 clusters and granola and were excluded from analysis; ^b^ nutrient profiling score (NPS) information was unavailable for 5 children’s cereals, 12 flakes, bubbles and puffs, 7 mueslis, 3 clusters and granola and were excluded from the analysis; ^c^ health star rating (HSR) information was unavailable for 4 children’s cereals, 44 flakes, bubbles and puffs, 60 mueslis, 30 clusters and granola and were excluded from the analysis.

**Table 2:** Nutrient comparison between children’s cereals with and without specific nutrient content and health claims.

Nutrient	Claim Present	Claim Not Present	*p*-Value
	Median (IQR)		
Wholegrain claim	*n* = 10	*n* = 23	
Energy (kJ)	1555 (103)	1600 (50)	0.062
Protein (g)	8.5 (3.3)	7.0 (2.2)	0.28
Fat (g)	2.2 (2.8)	1.2 (1.1)	0.025
Saturated fat (g)	1.0 (0.8)	0.3 (0.6)	0.31
Carbohydrate (g)	73.9 (5.5)	83.1 (10.3)	0.002
Sugar (g)	23.7 (10.8)	27.9 (18.8)	0.43
Sodium (mg)	123 (172)	312 (270)	0.008
Dietary fibre ^a^ (g)	8.4 (3.3)	2.5 (3.4)	0.001
Nutrient profiling score ^b^	1.0 (4.0)	9.0 (13.0)	0.018
Health star rating ^c^	4.0 (0.8)	2.5 (1.5)	0.001
Dietary fibre claim	*n* = 9	*n* = 24	
Energy (kJ)	1580 (85)	1595 (58)	0.46
Protein (g)	8.8 (1.7)	7.0 (2.1)	0.011
Fat (g)	2.5 (3.4)	1.2 (1.1)	0.011
Saturated fat (g)	1.0 (0.6)	0.3 (0.6)	0.006
Carbohydrate(g)	72.0 (6.0)	83.1 (9.8)	<0.001
Sugar (g)	25.1 (6.8)	28.0 (20.1)	0.59
Sodium (mg)	120 (115)	316 (264)	0.006
Dietary fibre ^a^ (g)	8.2 (2.5)	2.5 (4.8)	0.004
Nutrient profiling score ^b^	1.0 (3.0)	10.0 (13.0)	0.028
Health star rating ^c^	4.0 (0.8)	2.5 (1.5)	0.002
B Vitamins claim	*n* = 16	*n* = 17	
Energy (kJ)	1605 (65)	1580 (79)	0.87
Protein (g)	7.4 (3.5)	7.1 (2.3)	0.36
Fat (g)	1.5 (1.3)	0.9 (1.6)	0.049
Saturated fat (g)	0.8 (0.7)	0.2 (0.5)	0.031
Carbohydrate (g)	79.0 (11.6)	81.6 (14.1)	0.61
Sugar (g)	25.2 (17.2)	27.9 (17.5)	0.47
Sodium (mg)	277 (218)	150 (283)	0.47
Dietary fibre ^a^ (g)	4.4 (7.3)	7.1 (6.3)	0.87
Nutrient profiling score ^b^	4.0 (9.0)	2.0 (16.0)	0.98
Health star rating ^c^	3.0 (1.6)	3.5 (2.0)	0.85
Any claim	*n* = 25	*n* = 8	
Energy (kJ)	1590 (70)	1595 (70)	0.92
Protein (g)	7.8 (2.7)	6.1 (3.1)	0.12
Fat (g)	1.5 (1.8)	0.4 (0.6)	0.001
Saturated fat (g)	0.8 (0.7)	0.2 (0.2)	0.007
Carbohydrate (g)	77.4 (11.2)	86.3 (9.9)	0.008
Sugar (g)	25.1 (14.1)	32.3 (18.8)	0.067
Sodium (mg)	235 (225)	380 (334)	0.044
Dietary fibre ^a^ (g)	7.5 (6.6)	2.4 (5.7)	0.089
Nutrient profiling score ^b^	2.0 (8.0)	13.5 (12.0)	0.021
Health star rating ^c^	3.5 (1.3)	2.0 (1.5)	0.032

^a^ Dietary fibre information was unavailable for some products (five without wholegrain claim, five without fibre claim, four with B vitamins claim, one without B vitamins claim, five without any claim); ^b^ excluded five products from NPS analysis due to missing dietary fibre values; ^c^ excluded four products from HSR analysis as it was not displayed.
